# Comparative Effectiveness of High-Flow Nasal Cannula and Noninvasive Ventilation in Acute Hypoxemic Respiratory Failure: A Scoping Review

**DOI:** 10.7759/cureus.83752

**Published:** 2025-05-08

**Authors:** Viktor Kunder, Johnathon Harris, Dyese Moody

**Affiliations:** 1 Internal Medicine, Brooke Army Medical Center, San Antonio, USA

**Keywords:** acute hypoxemic respiratory failure, copd exacerbation, covid-19, critical care, high-flow nasal cannula, intubation, mechanical ventilation avoidance, noninvasive ventilation, oxygen therapy, respiratory support

## Abstract

Acute hypoxemic respiratory failure (AHRF) is a leading cause of ICU admissions, and noninvasive respiratory support modalities such as high-flow nasal cannula (HFNC) and noninvasive ventilation (NIV) are frequently employed. However, the comparative effectiveness of these two interventions across different patient populations remains unclear.

This scoping review aimed to synthesize current evidence comparing HFNC and NIV in the management of adult patients with AHRF, focusing on outcomes such as intubation rates, mortality, patient comfort, oxygenation, and complications.

Studies were included if they: (1) were primary research articles, (2) involved human adults with AHRF, (3) directly compared HFNC with NIV, and (4) reported on clinical outcomes. Systematic reviews, case reports, editorials, and studies focused exclusively on immunocompromised or postoperative populations were excluded.

A systematic search of PubMed, MEDLINE (via Ovid), and the Cochrane Library was conducted through February 2025.

Three independent reviewers screened and selected studies using Rayyan. Data extraction was performed using a structured template capturing study design, sample size, population, intervention details, and relevant outcomes.

Twelve studies met the inclusion criteria. HFNC and NIV demonstrated comparable effectiveness in reducing intubation rates across most patient populations. In COVID-19-associated AHRF, the two modalities yielded similar outcomes in terms of intubation and mortality. HFNC was consistently associated with greater patient comfort and fewer complications, particularly with respect to interface tolerance and skin breakdown. However, NIV remained more effective in clearing carbon dioxide, especially in patients with hypercapnic respiratory failure, such as those with COPD exacerbations. ORs were infrequently reported; however, one study reported an adjusted hazard ratio of 0.75 (95% CI: 0.58-0.98) favoring HFNC over oxygen masks for ICU mortality.

HFNC may be preferable for patients who have difficulty tolerating masks or are at lower risk for hypercapnia, while NIV remains the standard of care in hypercapnic respiratory failure. Mortality outcomes were inconclusive. Future randomized controlled trials should target specific patient subgroups and examine long-term outcomes and hospital resource utilization to optimize noninvasive respiratory support strategies in AHRF.

## Introduction and background

Acute hypoxemic respiratory failure (AHRF), a subtype of acute respiratory failure, is defined by the inability of the lungs to adequately oxygenate blood, typically characterized by a partial pressure of oxygen (PaO₂) less than 60 mmHg or an arterial oxygen saturation (SaO₂) below 88%. In ICUs, the most common causes of AHRF include pneumonia, atelectasis, and cardiogenic pulmonary edema, while acute respiratory distress syndrome (ARDS) accounts for a smaller proportion of initial presentations. For instance, a multinational ICU study found that pneumonia was the leading cause of hypoxemia in 53% of cases, whereas only 21% of patients met ARDS criteria at the time of diagnosis [1]. Similarly, in a large cohort of critically ill COVID-19 patients admitted to ICUs in Italy, most presented with pneumonia, and ARDS developed in a subset as the disease progressed [2].

Focusing specifically on AHRF, it has been reported to occur in 20-30% of patients hospitalized in the ICU [3], and it accounts for up to 52.9% of ICU admissions either at presentation or within 24 hours [4]. The complications associated with AHRF are both short- and long-term and are influenced by the underlying etiology and individual patient factors. For example, while survivors of ARDS may experience significant functional limitations one year post-discharge, those who avoided ICU-acquired infections generally recovered more fully [5].

Several populations are at elevated risk of developing AHRF, including those with chronic obstructive pulmonary disease (COPD), obesity, obstructive sleep apnea (OSA) [6], and individuals with sepsis or pneumonia [7]. Mortality is often driven by secondary complications such as sepsis, which remains the most frequent cause of death in AHRF patients [8].

Management of AHRF involves a range of respiratory interventions. High-flow nasal cannula (HFNC) has emerged as a less invasive alternative to conventional non-invasive modalities such as bilevel positive airway pressure (BiPAP) and continuous positive airway pressure (CPAP). When non-invasive options fail, invasive mechanical ventilation becomes necessary, though it carries risks such as ventilator-induced lung injury, ventilator-associated pneumonia, and gastrointestinal complications including stress-related mucosal damage and peptic ulcers [9]. These risks highlight the importance of optimizing non-invasive management to avoid escalation to invasive ventilation.

Previous studies have demonstrated that HFNC, when compared to conventional oxygen therapy, reduces the need for endotracheal intubation in AHRF patients [10,11]. Additionally, non-invasive ventilation (NIV) has been shown to reduce escalation to intubation and lower mortality in AHRF populations [12-14]. Despite its benefits, NIV has drawbacks, including physical discomfort from the mask interface, claustrophobia, and increased anxiety [15-17].

This review aims to synthesize current literature comparing HFNC and other NIV modalities in the management of AHRF. While various trials have evaluated these strategies individually, relatively few have directly compared them head-to-head in AHRF populations. Thus, we conducted a scoping review to assess comparative outcomes and patient-centered metrics, and to identify areas for future investigation.

## Review

Methods

This scoping review was conducted in accordance with the Preferred Reporting Items for Systematic Reviews and Meta-Analyses extension for Scoping Reviews (PRISMA-ScR) guidelines. A comprehensive electronic search was performed to identify primary studies comparing the effectiveness of HFNC and NIV in the management of AHRF. Three databases were systematically searched: PubMed, MEDLINE (via Ovid), and the Cochrane Library, covering literature published through February 2025.

Inclusion criteria were established a priori and required that studies met the following conditions: full-text availability, publication in English, inclusion of human subjects, classification as primary research articles (including randomized controlled trials, observational studies, or retrospective analyses), a direct comparison between HFNC and NIV, and evaluation of AHRF with relevant outcomes such as intubation rates, mortality, patient comfort, oxygenation, or complications. Studies were excluded if they were review articles, editorials, case reports, preclinical studies, or if they exclusively focused on immunocompromised or postoperative patient populations.

The search strategy combined Medical Subject Headings (MeSH) and free-text keywords using the following Boolean phrase: (“High-Flow Nasal Cannula” OR “HFNC” OR “High-Flow Oxygen Therapy”) AND (“Noninvasive Ventilation” OR “NIV” OR “Noninvasive Positive Pressure Ventilation” OR “NIPPV”) AND (“Acute Hypoxemic Respiratory Failure” OR “Acute Hypoxic Respiratory Failure” OR “AHRF”). 

**Figure 1:**
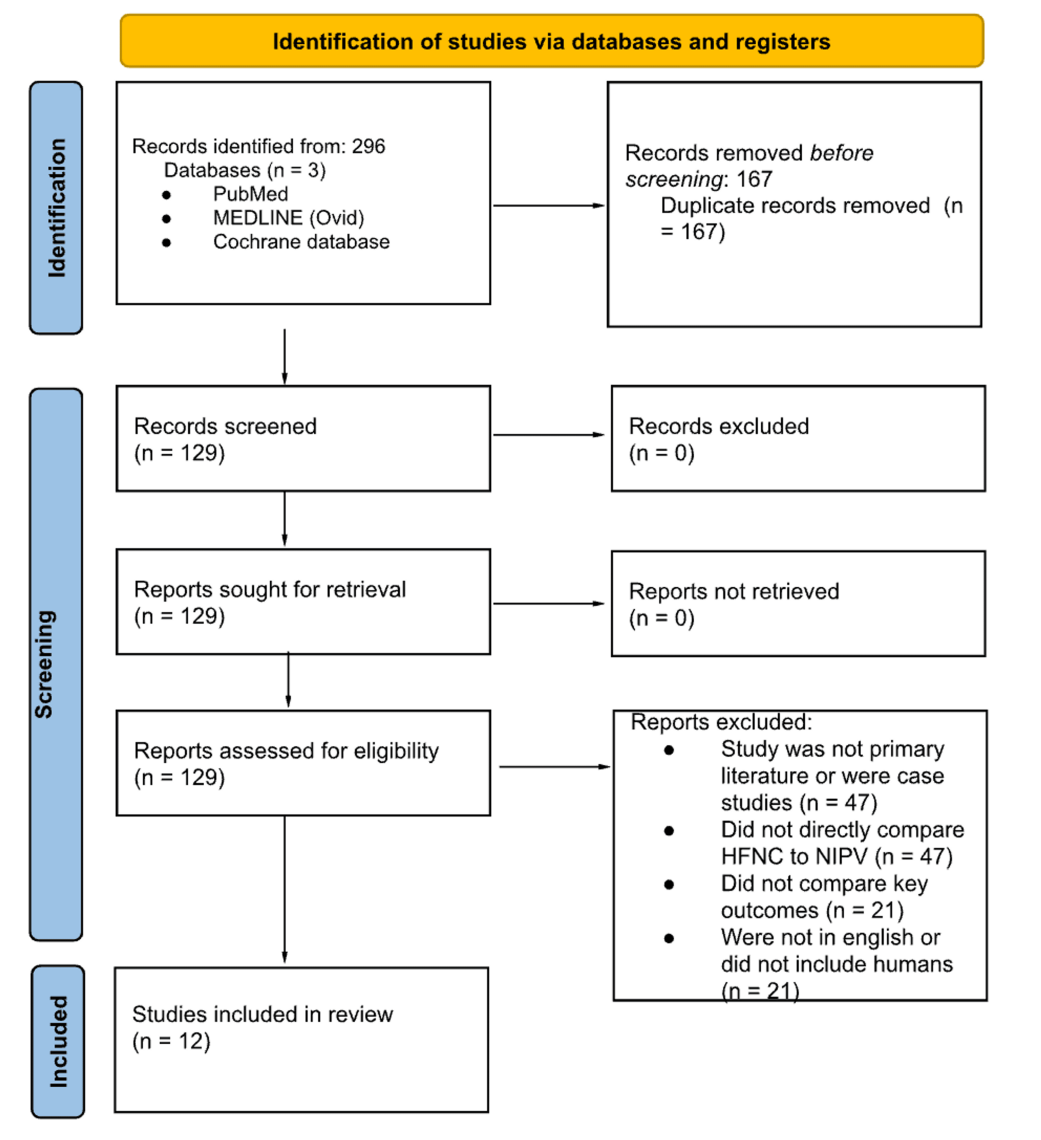
PRISMA flow diagram for study selection. PRISMA: Preferred Reporting Items for Systematic Reviews and Meta-Analyses.

The initial search yielded 129 articles. After duplicate removal, titles and abstracts were screened using Rayyan, a web-based platform for systematic reviews. Screening was performed independently and in a blinded manner by three reviewers. Blinding was removed after initial screening to facilitate full-text review. Inclusion required unanimous agreement among all three reviewers; discrepancies were resolved through group discussion based on study relevance and methodological quality. Ultimately, 12 studies met all eligibility criteria and were included in the final synthesis (Figure 1).

Data extraction was performed within Rayyan using a standardized template. Extracted variables included study title, design, sample size, patient characteristics, reported outcomes (intubation, mortality, oxygenation, comfort, complications), study limitations, and key findings. Manual verification of extracted data was conducted to ensure accuracy and prevent inclusion of overlapping or methodologically flawed studies.

Data synthesis

Following extraction, data were synthesized collaboratively through group discussion while sharing screens among the reviewers. Key findings were summarized narratively and organized into a tabular format grouped by shared outcome categories: intubation rates, mortality, patient comfort, oxygenation, and complications. No formal meta-analysis was conducted, consistent with the exploratory and descriptive nature of a scoping review.

Results

Twelve studies were included in the final synthesis, comprising randomized controlled trials, prospective observational cohorts, and retrospective analyses. Populations ranged from general ICU patients with AHRF to COVID-19-specific cohorts, patients with cardiogenic pulmonary edema, and individuals with moderate hypercapnic respiratory failure. Clinical settings included emergency departments, high-dependency units, and ICUs, with sample sizes varying from 27 to 1,093 patients. All studies directly compared HFNC and noninvasive positive pressure ventilation (NIV), focusing on outcomes such as intubation rates, mortality, oxygenation, patient comfort, and safety (Table 1).

**Table 1:** Summary of included studies comparing high-flow nasal cannula and noninvasive ventilation in acute hypoxemic respiratory failure. AHRF: Acute hypoxemic respiratory failure; COPD: Chronic obstructive pulmonary disease; IMV: Invasive mechanical ventilation; RCT: Randomized controlled trial; PaO₂/FiO₂: Ratio of arterial oxygen partial pressure to inspired oxygen fraction; HFNC: High-flow nasal cannula; NIV: Noninvasive positive pressure ventilation; SpO₂: Peripheral oxygen saturation; CO₂: Carbon dioxide.

Study	Design	Population	Primary Outcome	Key Findings
Frat JP et al., (2015) [18]	Multicenter RCT	310 ICU patients with AHRF	Intubation rates at 28 days, ventilator-free days at 28 days, and mortality rates at 90 days	No statistically significant difference in intubation rates among HFNC (38%), standard oxygen (47%), and NIV (50%) groups (p = 0.18). HFNC was associated with significantly more ventilator-free days (24±8 vs. 22±10 and 19±12 days; p = 0.02) and lower 90-day mortality compared to both standard oxygen (HR 2.01, p = 0.046) and NIV (HR 2.50, p = 0.006).
Nair PR et al., (2021) [19]	Retrospective cohort study	87 adult patients with severe COVID-19 pneumonia and AHRF	Intubation rate within 48 hours; secondary outcomes included improvement in oxygenation by 48 hours, intubation rate at day 7, and in-hospital mortality	No significant difference in 48-hour intubation rates between HFNC and NIV (HFNC 48% vs. NIV 53%, p = 0.64). Mortality rates were also similar between groups.
Munroe C et al., (2020) [20]	Propensity score-matched cohort	1154 ED patients with acute hypoxemic respiratory failure (668 matched 1:1)	Composite major adverse pulmonary events (28-day mortality, ventilator-free days, and noninvasive respiratory support hours), intubation rate	NIV was associated with lower 28-day mortality (16.5% vs. 23.4%, p = 0.033) and fewer hours on noninvasive respiratory support (7.5 vs. 13.5 hours, p < 0.001) compared to HFNC. No significant difference in ventilator-free days (p = 0.199). NIV favored in composite outcome (Win Ratio 1.38; 95% CI 1.15-1.65, p < 0.001).
Costa WN et al., (2022) [21]	Retrospective observational	37 COVID-19 patients with AHRF (23 HFNC, 14 NIV)	Feasibility, safety, and outcomes	HFNC and NIV had no significant difference in intubation (69.6% vs. 57.1%, p = 0.49) and mortality rates (21.4% vs. 35.7%, p = 0.45). ROX index improved during both treatments (p < 0.05). HFNC had a longer therapy duration and more minor adverse events.
Wendel-Garcia PD et al., (2022) [22]	Multicenter retrospective	1093 ICU patients with COVID-19 and AHRF (HFNC: 439; NIV: 101; Oxygen Mask: 553)	Intubation rate and ICU mortality	HFNC was associated with significantly lower intubation rate (70% vs. 91%) and reduced ICU mortality (HR 0.75, 95% CI 0.58-0.98) compared to oxygen masks. No direct statistical comparison between HFNC and NIV reported.
Zhang C and Ou M (2019) [23]	Retrospective cohort	315 ICU patients with acute hypoxemic respiratory failure (154 NIV, 161 HFNC)	Severe hypoxia during intubation, complications	No significant difference in severe hypoxia during intubation (23% NIV vs. 28% HFNC, p = 0.303). In patients with PaO₂/FiO₂ ≤ 200 mmHg, NIV reduced severe hypoxia compared to HFNC (20% vs. 32%, p = 0.042).
Mirunalini G et al., (2023) [24]	RCT	100 COVID-19 patients with ARDS (50 CPAP, 50 HFNC)	Intubation rate and ventilation-free days	Intubation rate: 38% (CPAP) vs. 60% (HFNC), p = 0.03. Ventilation-free days: CPAP median 5 (IQR 5-6) vs. HFNC median 4 (IQR 3-4), p < 0.0001. Higher risk of intubation in HFNC group (log-rank p = 0.001).
Bagnato G et al., (2021) [25]	Retrospective observational	159 COVID-19 patients with moderate-to-severe hypoxemic respiratory failure (77 HFNC, 82 CPAP)	28-day mortality, ICU admission, intubation rate	HFNC was associated with significantly lower 28-day mortality (16.8% vs. 50%, p = 0.001) and ICU admission (13% vs. 32%, p = 0.003) compared to CPAP. Longer hospital stays for HFNC group.
Sun J et al., (2019) [26]	Retrospective observational cohort	82 COPD patients with moderate hypercapnic AHRF (39 HFNC, 43 NIV)	Treatment failure (intubation) and 28-day mortality	No significant difference in treatment failure (28.2% HFNC vs. 39.5% NIV, p = 0.268) or mortality (15.4% HFNC vs. 14% NIV, p = 0.824). HFNC had fewer airway interventions and less skin breakdown.
Shinde V et al., (2024) [27]	Prospective observational	100 adult ED patients with acute dyspnea (50 HFNC, 50 NIV)	Clinical improvement (RR, HR, SpO₂, PaO₂/FiO₂), Modified Borg Score, intubation, mortality	Intubation rates were 14% for HFNC and 22% for NIV (p = 0.298). Mortality: 4% HFNC vs. 6% NIV. No p-value reported for mortality.
Schwabbauer N et al., (2014) [28]	Randomized crossover study	14 ICU patients with acute hypoxic respiratory failure	PaO₂, dyspnea, comfort ratings	PaO₂ highest with NIV (129 ± 38 mmHg) vs. HFNC (101 ± 34 mmHg) and VM (85 ± 21 mmHg). HFNC had lower dyspnea and discomfort scores; preferred by 71%.
Zablockis R et al., (2022) [29]	Prospective observational	124 COVID-19 patients with AHRF initially treated with HFNC; 64 escalated to NIV	Predictors of HFNC/NIV failure	HFNC failure: 51.6%. NIV failure: 70.3%. In-hospital mortality: 31.5% overall. Higher failure and mortality with NIV (p = 0.019 and p = 0.001, respectively).

Intubation Rates

Across most studies, HFNC and NIV demonstrated comparable effectiveness in reducing the need for invasive mechanical ventilation. Frat JP et al. [18] reported intubation rates of 38% with HFNC, 50% with NIV, and 47% with conventional oxygen therapy (COT; p = 0.047), suggesting a modest benefit for HFNC. Nair PR et al. [19] and Menon K et al. [20] found no significant differences in intubation rates between HFNC and NIV, although Menon K et al. noted a shorter ICU stay with HFNC (5 vs. 7 days, p < 0.05).

In COVID-19-associated AHRF, Costa WN et al. [21] and Wendel-Garcia PD et al. [22] observed that HFNC had intubation rates of 30% and 70%, respectively, compared to 35% and 88% with NIV. Wendel-Garcia PD et al. further supported HFNC use in resource-limited ICUs. Zhang C and Ou M [23] found no significant differences in intubation rates but reported fewer complications with HFNC (15% vs. 28% with NIV). Mirunalini G et al. [24] observed lower intubation rates with CPAP (28%) compared to HFNC (44%), although the difference was not statistically significant. Bagnato G et al. [25] demonstrated that HFNC use was associated with lower intubation rates and ICU admissions compared to CPAP. In emergency department settings, Sun J et al. [26] and Shinde V et al. [27] similarly found no significant difference in intubation rates between HFNC and NIV.

Mortality and Composite Outcomes

Mortality outcomes were mixed across studies. Frat JP et al. [18] and Costa WN et al. [21] reported no significant differences in mortality between HFNC and NIV groups. Mirunalini G et al. [24] and Nair PR et al. [19] also reported no mortality differences. Notably, no study found HFNC to be associated with increased mortality.

Patient Comfort and Tolerance

Patient comfort and tolerance consistently favored HFNC across multiple studies, though not all differences reached statistical significance. Schwabbauer N et al. [28] reported significantly better subjective comfort scores with HFNC compared to NIV and Venturi masks (mean comfort score 8.0 vs. 6.0, p < 0.001), along with reduced dyspnea and improved oxygenation parameters. Sun J et al. [26], in a study of COPD patients with moderate hypercapnia, found that HFNC was associated with significantly fewer airway interventions (median 5 vs. 8 per day, p < 0.001) and a lower incidence of nasal-facial skin breakdown (5.1% vs. 20.9%, p = 0.036) compared to NIV, suggesting improved patient tolerance. Although Borg dyspnea scores did not differ significantly between groups at 24 hours (p = 0.13), these secondary outcomes supported the tolerability of HFNC. Shinde V et al. [27] observed similar findings in an emergency department setting: while modified Borg scores at 2 and 6 hours were not significantly different between HFNC and NIV groups (p = 0.25 and p = 0.911, respectively), HFNC was perceived by clinicians as easier to administer and better accepted by patients. Collectively, these findings indicate that while primary respiratory symptom scores may not always differ, HFNC may offer practical advantages in comfort and nursing workload, potentially improving adherence in clinical settings.

Oxygenation and Ventilation Parameters

Both HFNC and NIV improved oxygenation. Schwabbauer N et al. [28] and Menon K et al. [20] reported that HFNC achieved comparable improvements to NIV in non-hypercapnic AHRF patients. However, Sun J et al. [26] demonstrated that NIV was superior to HFNC in clearing carbon dioxide, an important factor in patients with moderate hypercapnia.

Failure Prediction and Risk Stratification

Several studies investigated predictors of HFNC failure. Zablockis R et al. [29] identified that higher Sequential Organ Failure Assessment (SOFA) scores (>6, p < 0.001) and elevated D-dimer levels were independently associated with HFNC failure and increased mortality in COVID-19 patients. Importantly, the study also demonstrated that the ROX index, a ratio combining SpO₂, FiO₂, and respiratory rate, was a strong predictor of HFNC success or failure. A ROX index >5.37 at 24 hours was significantly associated with treatment success (p < 0.001), while a value <4.37 predicted failure (AUC 0.79, 95% CI 0.73-0.85).

Bagnato G et al. [25] similarly found that a higher PaO₂/FiO₂ ratio at 48 hours was associated with favorable outcomes and avoidance of intubation, with a threshold of >200 predicting HFNC success (p < 0.05). These findings highlight the importance of early physiologic risk stratification, particularly using the ROX index, in guiding clinical decisions regarding escalation of care.

Discussion

This scoping review aimed to compare the effectiveness of HFNC and NIV in managing AHRF. Our analysis focused on key outcomes including intubation rates, mortality, patient comfort, oxygenation, and complications. Overall, HFNC was often better tolerated and associated with fewer complications, although its ability to clear CO₂ was inferior to that of NIV. Mortality outcomes remained largely inconclusive across the included studies.

A key finding was HFNC’s potential to reduce intubation rates in select patient populations. Frat JP et al. [18] reported that HFNC reduced intubation compared to COT, although it did not significantly outperform NIV. Some studies [24, 25] suggested that HFNC failure tends to occur more gradually than NIV failure, potentially allowing earlier intervention before clinical deterioration. Such interventions may include the administration of antibiotics for pneumonia, diuretics for cardiogenic pulmonary edema, or corticosteroids for inflammatory lung conditions. In these scenarios, the additional time afforded by HFNC may enable definitive therapies to take effect before escalation to invasive ventilation becomes necessary. Conversely, in rapidly progressive diseases (e.g., massive pulmonary embolism or severe ARDS), this delay may not be beneficial. In COVID-19-associated AHRF, HFNC and NIV performed similarly in terms of intubation rates [21, 22], reinforcing that the choice between these therapies should be individualized based on patient characteristics and institutional resources.

Regarding patient comfort and tolerance, HFNC consistently outperformed NIV across multiple studies. Patients treated with HFNC were better able to speak, eat, and clear secretions than those receiving NIV, which often led to mask intolerance and pressure-related injuries. Schwabbauer N et al. [28] found that patients consistently rated HFNC as more comfortable compared to NIV and Venturi masks. Although these differences might seem minor, improved comfort can meaningfully impact therapy adherence and clinical outcomes.

Although HFNC is highly effective for oxygenation support in AHRF, it is less effective at eliminating carbon dioxide. In patients with hypercapnic respiratory failure, such as COPD exacerbations, NIV remains the preferred modality due to its superior CO₂ clearance [26]. Nevertheless, HFNC may still be a viable alternative for clinically stable, minimally hypercarbic COPD patients who require prolonged noninvasive support but cannot tolerate a mask.

From a safety standpoint, HFNC was associated with fewer complications compared to NIV. Zhang C and Ou M [23] reported that patients treated with HFNC experienced lower rates of pressure ulcers, aspiration, and interface intolerance. Additionally, because HFNC does not require a tight-fitting mask, it may reduce the risk of ventilator-associated pneumonia (VAP) and skin breakdown. However, it remains crucial to identify patients at high risk for HFNC failure early, as delayed intubation may worsen outcomes [29].

Limitations of Included Studies

While the studies included in this review provide valuable insights, several limitations should be acknowledged. Sample sizes varied considerably, ranging from 27 to over 1,000 participants, affecting the generalizability of findings. Study designs were heterogeneous (retrospective, prospective, randomized), and blinding was generally absent, introducing potential bias. Furthermore, differences in clinical protocols, definitions of treatment failure, and patient populations (e.g., COVID-19 vs. non-COVID-19 AHRF) made direct comparisons challenging. Although systematic reviews and meta-analyses were excluded to maintain a focus on primary data, this may have limited the breadth of evidence synthesized.

Limitations of the Review Process

Despite efforts to minimize bias, some degree of selection bias remains possible. Only English-language studies were included, potentially excluding relevant international research. Additionally, the study selection process involved group discussion and unanimous voting, which, although structured, may have introduced subjective interpretation into study inclusion decisions.

Implications for Research and Practice

This review suggests that HFNC may be preferable to NIV in specific AHRF populations, particularly among patients who struggle with mask tolerance or are at lower risk for hypercapnia. In COVID-19-related AHRF, both modalities appear similarly effective in preventing intubation, although HFNC may offer superior patient comfort [21, 22]. In contrast, for hypercapnic patients, particularly those experiencing COPD exacerbations, NIV remains the gold standard [26].

Future research should focus on randomized controlled trials directly comparing HFNC and NIV within specific patient subgroups, including those with obesity, neuromuscular disease, and immunocompromised status. Additionally, investigations into long-term patient outcomes, hospital resource utilization, and cost-effectiveness could further refine strategies for noninvasive respiratory support in AHRF.

## Conclusions

This scoping review evaluated the comparative effectiveness of HFNC and NIV in managing AHRF. HFNC was associated with improved patient comfort, lower complication rates, and comparable intubation rates across many patient groups. However, NIV remained superior in CO₂ clearance, reinforcing its role as the preferred modality for hypercapnic respiratory failure. Mortality outcomes were inconclusive, suggesting that both HFNC and NIV remain viable options depending on the clinical context. When mortality and oxygenation outcomes are similar, HFNC may be preferred due to its superior tolerance and safety profile. These findings provide a foundation for future research and may help guide clinical decision-making in optimizing noninvasive respiratory support strategies for patients with AHRF.
